# Recalibration of the ACC/AHA Risk Score in Two Population-Based German Cohorts

**DOI:** 10.1371/journal.pone.0164688

**Published:** 2016-10-12

**Authors:** Tonia de las Heras Gala, Marie Henrike Geisel, Annette Peters, Barbara Thorand, Jens Baumert, Nils Lehmann, Karl-Heinz Jöckel, Susanne Moebus, Raimund Erbel, Christine Meisinger, Amir Abbas Mahabadi, Wolfgang Koenig, on behalf of the KORA Study Group and the Heinz Nixdorf Recall Study Group

**Affiliations:** 1 Institute of Epidemiology II, Helmholtz Zentrum München, German Research Center for Environmental Health, Neuherberg, Germany; 2 German Centre for Cardiovascular Research (DZHK), partner site Munich Heart Alliance, Munich, Germany; 3 Institute of Medical Informatics, Biometry and Epidemiology, University of Duisburg-Essen, Essen, Germany; 4 Department of Cardiology, The West-German Heart and Vascular Center, University of Duisburg-Essen, Essen, Germany; 5 Central Hospital of Augsburg, MONICA/KORA Myocardial Infarction Registry, Augsburg, Germany; 6 Department of Internal Medicine II- Cardiology, University of Ulm, Medical Center, Ulm, Germany; 7 Deutsches Herzzentrum München, Technische Universität München, Munich, Germany; Erasmus Universiteit Rotterdam, NETHERLANDS

## Abstract

**Background:**

The 2013 ACC/AHA guidelines introduced an algorithm for risk assessment of atherosclerotic cardiovascular disease (ASCVD) within 10 years. In Germany, risk assessment with the ESC SCORE is limited to cardiovascular mortality. Applicability of the novel ACC/AHA risk score to the German population has not yet been assessed. We therefore sought to recalibrate and evaluate the ACC/AHA risk score in two German cohorts and to compare it to the ESC SCORE.

**Methods:**

We studied 5,238 participants from the KORA surveys S3 (1994–1995) and S4 (1999–2001) and 4,208 subjects from the Heinz Nixdorf Recall (HNR) Study (2000–2003). There were 383 (7.3%) and 271 (6.4%) first non-fatal or fatal ASCVD events within 10 years in KORA and in HNR, respectively. Risk scores were evaluated in terms of calibration and discrimination performance.

**Results:**

The original ACC/AHA risk score overestimated 10-year ASCVD rates by 37% in KORA and 66% in HNR. After recalibration, miscalibration diminished to 8% underestimation in KORA and 12% overestimation in HNR. Discrimination performance of the ACC/AHA risk score was not affected by the recalibration (KORA: C = 0.78, HNR: C = 0.74). The ESC SCORE overestimated by 5% in KORA and by 85% in HNR. The corresponding C-statistic was 0.82 in KORA and 0.76 in HNR.

**Conclusions:**

The recalibrated ACC/AHA risk score showed strongly improved calibration compared to the original ACC/AHA risk score. Predicting only cardiovascular mortality, discrimination performance of the commonly used ESC SCORE remained somewhat superior to the ACC/AHA risk score. Nevertheless, the recalibrated ACC/AHA risk score may provide a meaningful tool for estimating 10-year risk of fatal and non-fatal cardiovascular disease in Germany.

## Introduction

In 2013, the American College of Cardiology and the American Heart Association (ACC/AHA) released new guidelines on the diagnosis and treatment of elevated blood cholesterol and an equation for the assessment of cardiovascular risk in the American population, defined as the risk of a first arteriosclerotic cardiovascular disease (ASCVD) event in 10 years.[1, 2] While the ACC/AHA risk score considers non-fatal myocardial infarction, coronary heart disease death, as well as fatal or non-fatal stroke, the in Europe established European Society of Cardiology risk score [3] (ESC SCORE) only estimates risk of cardiovascular mortality.

External validations of the ACC/AHA risk score have been conducted in several U.S. cohorts [4–6] and in two European populations [7, 8] and substantial overestimation has been reported.[4–7] However, its performance has neither been tested in a German population nor has the ACC/AHA risk score been recalibrated to meet the underlying ASCVD risk in Germany.

We therefore sought to evaluate the ACC/AHA risk score prospectively in two population-based German cohorts with different background risk for ASCVD, the Monitoring of Trends and Determinants in Cardiovascular Disease (MONICA)/Cooperative Health Research in the Region of Augsburg (KORA) and the Heinz Nixdorf Recall (HNR) Studies. We recalibrated the ACC/AHA risk score using these German cohorts and compared performance of the original risk score with its recalibrated version and with the ESC SCORE.

## Methods

### Study populations

#### Cooperative Health Research in the Region of Augsburg Study

The KORA S3 and S4 surveys are population-based studies with baseline examinations in 1994–1995 and 1999–2001, respectively, including subjects from the city of Augsburg and the two adjacent counties located in the state of Bavaria in Southern Germany. Lifestyle factors and health conditions were assessed in computer-assisted personal interviews. Physical examinations were conducted by trained medical staff. Non-fatal stroke and non-fatal myocardial infarction were assessed by follow-up questionnaires in 2009 and validated by chart reviews or by contacting the subject`s primary care physician. Events of fatal stroke and fatal myocardial infarction were identified in 2011 by death certificates. In addition, information on non-fatal and fatal myocardial infarction was updated with information from the MONICA/KORA Myocardial Infarction Registry [9]. Subjects who had moved outside the study area were traced by address research; follow-up questionnaires were sent to their new address and self-reported non-fatal stroke or non-fatal myocardial infarction were validated by consulting the responsible local hospital. Detailed information on the KORA studies has been published elsewhere.[10]

**Fig 1:**
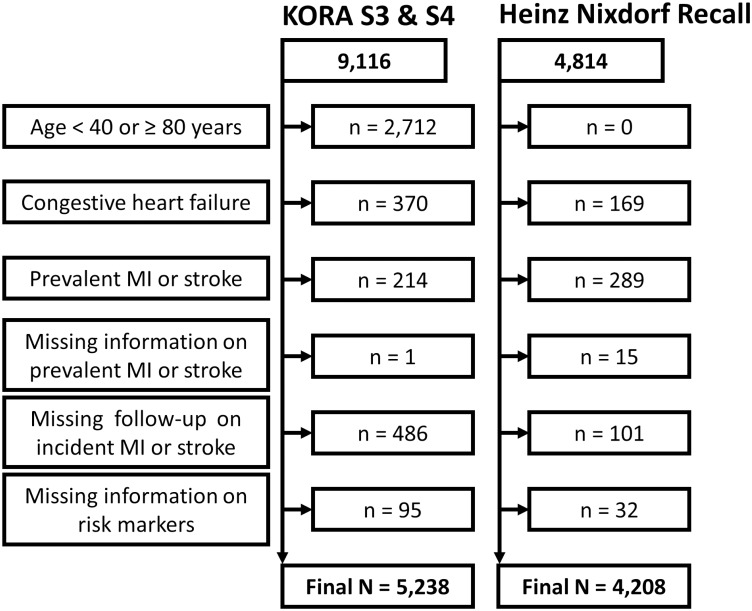
Flow chart of study populations. Flow chart of the KORA and Heinz Nixdorf Recall study populations showing exclusion criteria in accordance with the ACC/AHA risk score.

For calculating the ACC/AHA risk score and in accordance with its empirical derivation,[2] subjects were excluded if they were outside the predefined age range of 40–79 years or had a previous history of congestive heart failure, myocardial infarction, or stroke prior to baseline. In addition, we excluded subjects with missing information on prevalent or incident events of myocardial infarction and stroke, as well as subjects with missing information on risk markers that were necessary to calculate the ACC/AHA risk score. Unlike Goff et al.[2], we were not able to exclude subjects with previous unrecognized myocardial infarction, percutaneous coronary intervention (PCI), coronary bypass surgery or atrial fibrillation, due to incompleteness or unavailability of this information in KORA S3 and S4. With these criteria, out of 9,116 KORA S3 and S4 subjects, 3,878 subjects had to be excluded, resulting in a final study population of 5,238 subjects (for details see Fig 1).

In KORA, 486 subjects were lost-to-follow-up without information on incident ASCVD. To rule out possible bias in analysing the ACC/AHA risk score, we performed a drop-out analysis. Detailed description and results are shown in S1 Appendix.

#### Heinz Nixdorf Recall Study

The Heinz Nixdorf Recall study is a population-based prospective cohort study, designed to assess the predictive value of novel risk markers in addition to traditional cardiovascular risk factors. Participants were randomly selected from mandatory lists of residence from the three adjacent cities of Bochum, Essen and Mülheim, located in the Rhine-Ruhr-Area in Western Germany and enrolled between 2000 and 2003. Details of recruitment, study design, and risk factor assessment have been previously published.[11–13] Annually, questionnaires on the current state of health were sent out to all participants. In parallel, death certificates were regularly screened. Incident coronary events (myocardial infarction, sudden cardiac death and fatal coronary heart disease) and strokes were validated by review of all available hospital records and records of the attending physicians and adjudicated by an external endpoint committee. Fatal or non-fatal myocardial infarction was defined based on symptoms, electrocardiographic signs, cardiac enzymes, and necropsy. Stroke was defined as a focal neurological deficit over a period of >24hours of presumed cerebrovascular origin. In addition, cardiovascular mortality of any cause was classified by the statistical state office based on death certificate information according to the International Statistical Classification of Disease (ICD).

Exclusion criteria were applied according to those in KORA. From an overall HNR population of 4,814 subjects, 606 had to be excluded (thereof 101 without information on incident myocardial infarction or stroke), resulting in 4,208 eligible subjects (for details see Fig 1).

#### Ethical standards

All subjects signed written informed consent. For KORA, study protocols have been approved by the local Ethics Committee of the Bavarian Medical Association. The research protocol of the Heinz Nixdorf Recall study has been approved by the Ethics Committee of the Medical Faculty, University Duisburg-Essen.

### The ACC/AHA risk score

The ACC/AHA risk score consists of sex- and race-specific equations estimating the individual 10-year risk of hard ASCVD events, defined as the first occurrence of a non-fatal myocardial infarction, coronary heart disease death, fatal or non-fatal stroke.[2] Risk factors included in the risk equations are age (years), total cholesterol (mg/dL), high-density lipoprotein (mg/dL), systolic blood pressure (mm Hg), blood pressure treatment (yes/no), current smoking (yes/no) and diabetes (yes/no). The ACC/AHA guidelines include different equations for Caucasians and non-Caucasians, hence, we used the equations for Caucasians for risk estimation in the two German cohorts. Details on the calculation of the ACC/AHA risk equations were derived from Goff et al. [2] An online calculator is available on http://www.cvriskcalculator.com/.

To consider differing background risk of the U.S. and Germany, we recalibrated the original ACC/AHA risk equations by Goff et al. [2] Using the pooled data set of the two German cohorts, we applied the recalibration technique described in Janssen et al., which corrects the risk score to comply with ‘calibration in the large’ [14]. This method calibrates the estimated risk values by adding the correction factor $\text{ln}\left(\frac{\frac{observed\;event\;frequency}{1-observed\;event\;frequency}}{\frac{mean\;predicted\;risk}{1-mean\;predicted\;risk}}\right)$ to the linear predictor within the risk equation. The original and recalibrated risk equations are summarized in S1 Table.

### The ESC SCORE

Predicting 10-year risk of cardiovascular mortality, the ESC SCORE equation includes information on sex, age (years), total cholesterol (mmol/L), systolic blood pressure (mmHg) and current smoking (yes/no). Baseline survival was estimated separately for high-risk and low-risk countries. Since Germany is classified as a low-risk country, we used the corresponding equation. Calculations were performed as detailed in Conroy et al.[3]

For the ESC SCORE, events in the two study populations were re-categorized into fatal cardiovascular events with pre-determined ICD codes as defined in Conroy et al.[3] Exclusion criteria of the ESC SCORE differed from those of the ACC/AHA risk score and are illustrated in detail in S1 Fig.

### Statistics

If not indicated otherwise, continuous variables are presented as median (first quartile, third quartile). Categorical variables are given as absolute number (percentage). Incidence rates were calculated as the number of incident events divided by the sum of person-years of all subjects at risk. Follow-up times of more than 10 years were censored.

To evaluate the risk scores, we observed discrimination and calibration performance of the risk scores for the pooled cohorts as well as separately by cohort. Discrimination performance of the risk scores was evaluated using Harrell’s concordance statistic (C-statistic) for survival data.[15, 16] Agreement of estimated risk and observed event frequency was assessed by a calibration plot categorizing subjects in deciles of estimated risk and comparing mean estimated risk versus average incidence per decile.[17, 18] In addition, the degree of miscalibration was assessed using following formula: Overestimation (%) = [(Estimated risk rate / Observed event rate) - 1] * 100. Positive numbers indicate an overestimation of the observed event rate, negative numbers stand for an underestimation.

Results regarding calibration and discrimination performance of the recalibrated ACC/AHA risk score were validated using 10-fold cross-validation. This method of internal validation ensures an unbiased estimation of the performance measures.

Statistical analysis was done in R version 3.0.2 using packages pROC, survival and survcomp.[19–22]

## Results

### Characteristics of study cohorts

The KORA study population consisted of 5,238 subjects aged 56.0 ± 9.7 (mean ± standard deviation) years, and 49% men (Table 1). During an average follow-up time of 8.6 ± 2.0 years, 383 (7.3%) incident ASCVD events occurred. From the HNR study, 4,208 subjects aged 59.0 ± 7.7 years (48% men) could be included. Of these, 271 (6.4%) subjects had a first incident ASCVD event during an average follow-up time of 9.0 ± 2.1 years. Comparing the risk profile of the two cohorts, we observed small differences regarding intake of antihypertensive drugs, intake of statins or fibrates and prevalence of diabetes. Incidence rates of ASCVD were 8.5 and 7.1 per 1,000 person years in KORA and in HNR, respectively. In both cohorts, incidence of ASCVD was more than two times higher in men compared to women.

**Table 1:** Basic description of study populations after applying exclusion criteria according to the ACC/AHA risk score.

Characteristic^a^	KORA		HNR	
	Men	Women	Men	Women
**N (%)**	2,584 (49)	2,654 (51)	2,005 (48)	2,203 (52)
**Age**^b^ **(years)**	56.4 ± 9.6	55.5 ± 9.7	58.8 ± 7.6	59.1 ± 7.7
**Total cholesterol (mg/dL)**	235 (208, 263)	233 (209, 264)	226 (202, 249)	232 (208, 260)
**HDL cholesterol (mg/dL)**	48 (40, 58)	60 (49, 72)	49 (42, 59)	64 (53, 75)
**Systolic blood pressure (mmHg)**	136 (125, 149)	128 (116, 144)	137 (125, 149)	125 (113,141)
**Intake of antihypertensive drugs**	486 (19)	583 (22)	604 (30)	696 (32)
**Intake of statins or fibrates**^c^	111 (4)	138 (5)	180 (10)	178 (9)
**Current smoker**	639 (25)	457 (17)	518 (26)	474 (22)
**Diabetes**	136 (5)	115 (4)	167 (8)	127 (6)
**Incident ASCVD events**	257 (10)	126 (5)	186 (9)	85 (4)
**Fatal cardiovascular events**^d^	119 (4)	56 (2)	49 (3)	25 (1)

N, Sample size

HDL, High-density lipoprotein

ASCVD, atherosclerotic cardiovascular disease

^a^Depicted are absolute numbers (percentage) for categorical and median (first quartile, third quartile) for continuous variables.

^b^Age (years) is shown as mean ± standard deviation.

^c^In HNR, information on intake of fibrates or statins was only available in n = 1,861/2,071 men/women.

^d^Fatal cardiovascular events according to Conroy et al.[3] after applying regarding exclusion criteria (Sample size for KORA men/women n = 2,805/2,950 and for HNR men/women n = 1,929/2,158).

### Calibration

Table 2 provides, next to the observed and estimated event rates, a comparison of the magnitude of overestimation by the original and the recalibrated ACC/AHA risk score, as well as by the ESC SCORE.

**Table 2:** Overall overestimation by risk scores.

	KORA			HNR		
	Total	Men	Women	Total	Men	Women
**ACC/AHA risk score**						
**- Original**						
Observed frequency [%]	7.3	9.9	4.7	6.4	9.3	3.9
Estimated risk [%]	10.0	14.2	5.9	10.7	15.1	6.6
Overestimation [%]	36.9	42.6	25.1	65.9	63.2	71.7
**- Recalibrated**						
Observed frequency [%]	7.3	9.9	4.7	6.4	9.3	3.9
Estimated risk [%]	6.7	9.6	4.0	7.2	10.3	4.4
Overestimation [%]	-7.7	-3.3	-16.7	11.7	10.6	14.2
**ESC SCORE**						
Observed frequency [%]	3.0	4.2	1.9	1.8	2.5	1.2
Estimated risk [%]	3.2	4.5	2.0	3.3	4.7	2.1
Overestimation [%]	5.3	5.3	5.3	84.6	86.6	80.8

The original ACC/AHA risk score overestimated risk by 43% in men and 25% in women in KORA and by 63% in men and 72% in women in HNR, respectively. Recalibration of the ACC/AHA risk score based on the observed event rate in both cohorts led to a substantially reduced miscalibration resulting in an underestimation of risk by 3% in men and 17% in women in KORA and an overestimation of risk by 11% in men and 14% in women in the HNR cohort.

The ESC SCORE overestimated observed event rates in KORA by 5% in men as well as by 5% in women, while in HNR overestimation amounted to 87% in men and 81% in women. In KORA, risk estimates and observed event frequencies therefore agreed better using the ESC SCORE than using the original or recalibrated ACC/AHA risk score, whereas in the HNR population, the recalibrated ACC/AHA risk score was the best calibrated score.

**Fig 2:**
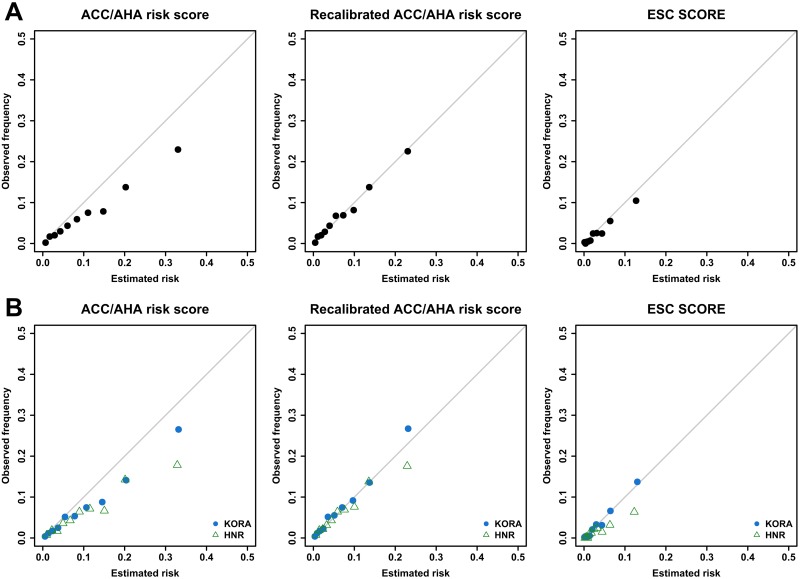
Calibration plots. Calibration plots of ACC/AHA risk score before (left side) and after (middle) recalibration and of ESC SCORE (right side). Part A shows calibration in the pooled sample of KORA and HNR. Part B shows calibration by cohort (KORA: filled circles, HNR: triangles). The solid line indicates perfect calibration of the risk score. Depicted are mean estimated risk versus mean observed frequency per decile of estimated risk with axes ranging from 0 to 0.5 (50%).

These findings were confirmed by the calibration plots (Fig 2) which give more insight into the complete risk range. For the pooled sample as well as separately by cohort, the calibration curves of the ACC/AHA risk score were deflected below the bisecting line, particularly in the highest risk deciles. The recalibrated ACC/AHA risk score showed considerably improved calibration over the complete risk range: for KORA, we detected slight underestimation and for HNR slight overestimation in the highest risk decile. The ESC SCORE revealed almost perfect calibration in KORA and overestimation in the upper risk categories in HNR.

As the KORA cohort consisted of two individual surveys (S3 and S4) from different recruitment periods, we also investigated calibration performance of the ACC/AHA risk score separately for both. Risk was overestimated by the original risk score in both surveys, however, considerably more in KORA S4. Recalibration reduced miscalibration for both surveys (S2 Fig).

### Discrimination

**Fig 3:**
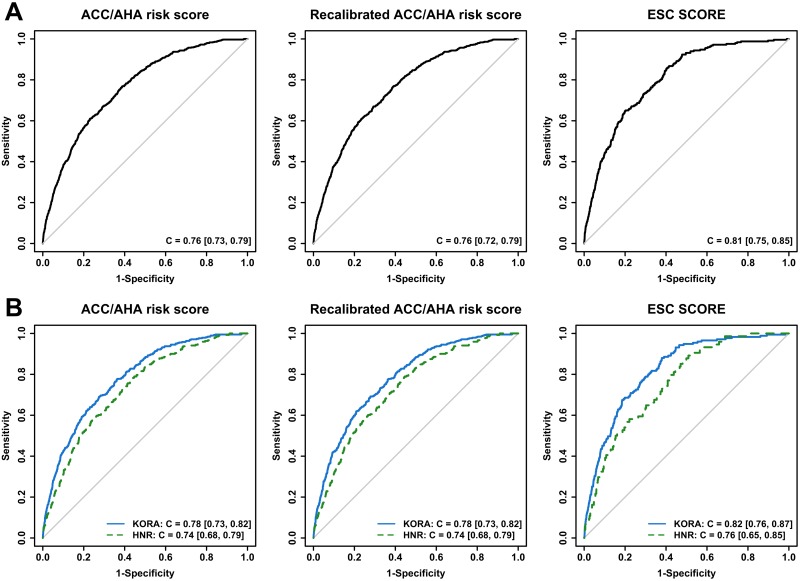
ROC curves. ROC curves of ACC/AHA risk score before (left side) and after (middle) recalibration and of ESC SCORE (right side). Part A illustrates performance in the pooled sample of KORA and HNR, while part B shows performance separately for KORA (solid) and HNR (dashed). C = C-statistic [95% confidence interval].

Discrimination analysis of the ACC/AHA risk score showed a C-statistic [95% confidence interval] of C = 0.76 [0.73, 0.79] in the pooled sample, C = 0.78 [0.73, 0.82] in the KORA cohort and C = 0.74 [0.68, 0.79] in the HNR cohort (Fig 3). As expected, these findings were identical to the discriminative performance of the recalibrated score. Estimating 10-year risk of cardiovascular mortality, the ESC SCORE showed better discrimination ability (pooled sample: C = 0.81 [0.75, 0.85], KORA: C = 0.82 [0.76, 0.87], HNR: C = 0.76 [0.65, 0.85]) than both the original and the recalibrated ACC/AHA risk score. All risk scores resulted in a better discriminative performance in KORA compared to HNR.

## Discussion

We found considerable overestimation of 10-year ASCVD risk by the original ACC/AHA risk score in two German population-based cohorts. This observation is in line with results from Kavousi et al. who detected consistent overestimation across the whole range of risk in a Dutch population.[7] Vaucher et al., who investigated the economic impact of the new ACC/AHA guidelines compared to the ESC guidelines in a Swiss population, observed a larger number of high-risk individuals when applying the ACC/AHA guidelines.[8] They suggested that differences in risk factor profiles between the U.S. and Europe could be the reason for this observation. Other studies found a similar overestimation when assessing the original Framingham risk score in European populations.[23–25] Given these observations, it is likely that the overestimation of risk that we found in our study when applying the original ACC/AHA score to a German population may at least in part be attributable to a different risk profile compared to the U.S population.

Nevertheless, we also have to consider the recruitment time of the studies included in the development of the ACC/AHA risk equations. Goff et al. incorporated study cohorts such as the Framingham original and offspring, the Atherosclerosis Risk in Communities (ARIC), the Coronary Artery Risk Development in Young Adults (CARDIA), and the Cardiovascular Health Study (CHS) cohort, most of which recruited their participants in the mid-1980s.[2] Changing risk profiles over time may contribute to risk overestimation when applying the ACC/AHA risk score to more contemporary populations.[26] This hypothesis is supported by our analysis wherein we found that the original ACC/AHA risk score performed best in the earliest recruited cohort KORA S3 (1994–95), had a poorer performance in the more recent cohort KORA S4 (1999–2001) and performed worst in HNR, the most recently assessed study population (2000–03). While diagnostic changes over time are one plausible explanation for such differences,[27] alterations in lifestyle factors and emerging risk markers, which may not have been sufficiently considered in the ACC/AHA risk score, would also explain the performance discrepancy of the ACC/AHA risk score.

We observed differences in the performance of the ACC/AHA risk score and the ESC SCORE between the KORA and the HNR study population. Different recruitment time points of HNR, KORA S3 and KORA S4 may be partly responsible for these differences, reflecting, in part, the decreasing incidence of cardiovascular events over the last 10 years.[28] In addition, we must consider regional diversity regarding cardiovascular risk within Germany.[29–31] We found a higher prescription rate of antihypertensives and lipid lowering drugs in HNR, possibly explaining the slightly lower incidence rate of ASCVD in the HNR population.

The different performance of the ESC SCORE in HNR and KORA is of particular interest, as an independent earlier survey within the KORA study (MONICA/KORA S1, 1984/85) contributed to the development of the ESC SCORE as the only population sample from Germany.[3] This may also explain the superior performance of the ESC SCORE in KORA compared to HNR. Nevertheless, such discrepancies in an established European risk score demonstrate the necessity of reconsidering the ESC SCORE regarding included risk factors and the requirement of including contemporary studies from Germany, such as HNR.

In our approach we focused on the recalibration of the ACC/AHA risk score, which means the evaluation and reassessment of the original risk score by using the originally included risk markers. However, recalibration does not consider the possibility of evaluating additional risk factors in the original score, such as family history of cardiovascular events. We would like to encourage future studies to include such risk factors to potentially increase the performance of the recalibrated ACC/AHA risk score.

Finally, we cannot rule out different ascertainment strategies for fatal and non-fatal ASCVD events in HNR and KORA as an additional possible source for the observed differences in the performance of the risk scores.

### Limitations

One minor limitation of this study is the incomplete follow-up (follow-up time of less than 8 years) on non-fatal stroke in n = 278 subjects of the KORA survey S4. Although we do have information on non-existence of myocardial infarction and fatal stroke events in these subjects, we may have missed non-fatal stroke events. However, considering the small number of non-fatal stroke events after 8 years of follow-up in KORA survey S3 (event rate 0.5%), we assume that this incomplete follow-up in KORA survey S4 did not affect our results considerably.

We were not able to exclude subjects with previous unrecognized myocardial infarction, percutaneous coronary intervention (PCI), coronary bypass surgery or atrial fibrillation as it was done in the original version of the ACC/AHA risk score. Although unlikely, it is possible that these exclusions might have improved performance of the uncalibrated risk equations.

Our study compares the ACC/AHA risk score with the ESC SCORE, however, we did not consider a comparison to the only other Germany-specific risk score, the Prospective Cardiovascular Münster (PROCAM) score. This score is based on data from employees of companies and local government authorities and offers risk equations separate for major coronary events and stroke.[32] Although the PROCAM score showed good performance within the PROCAM cohort, one must question whether the development of a risk score only based on employed individuals can be representative for the general German population. For this reason, we have not considered its incorporation in our analysis.

## Conclusions

The recalibrated ACC/AHA risk score showed improved risk prediction as compared to the original ACC/AHA risk score in both German cohorts. Predicting only cardiovascular mortality, discrimination performance of the commonly used ESC SCORE remained somewhat superior to the ACC/AHA risk score. Yet non-fatal events are of increasing importance given the declining case-fatality of acute myocardial infarction. Considering this advantage, the recalibrated ACC/AHA risk score provides a meaningful tool for estimating 10-year risk of fatal and non-fatal cardiovascular disease in the German population.

## Supporting Information

S1 Appendix Drop-out analysis. (PDF)

S1 Fig Flow chart of study populations showing exclusion criteria in accordance with the ESC SCORE. (PDF)

S2 Fig Calibration plot by KORA survey. Calibration plot of the ACC/AHA risk score before (left) and after (right) recalibration separately for KORA S3 (filled circles) and KORA S4 (triangles).
(PDF)

S1 Table Calculation of ACC/AHA risk score before and after recalibration. (PDF)

## References

[1] Stone NJ, Robinson JG, Lichtenstein AH, Bairey Merz CN, Blum CB, Eckel RH, et al 2013 ACC/AHA Guideline on the Treatment of Blood Cholesterol to Reduce Atherosclerotic Cardiovascular Risk in Adults: a Report of the American College of Cardiology/American Heart Association Task Force on Practice Guidelines. J Am Coll Cardiol. 2014;63(25 Pt B):2889–934. 10.1016/j.jacc.2013.11.002 24239923

[2] Goff DC Jr., Lloyd-Jones DM, Bennett G, Coady S, D'Agostino RB, Gibbons R, et al 2013 ACC/AHA Guideline on the Assessment of Cardiovascular Risk: A Report of the American College of Cardiology/American Heart Association Task Force on Practice Guidelines. Circulation. 2014;129(25 Suppl 2):S49–73. 10.1161/01.cir.0000437741.48606.98 24222018

[3] Conroy RM, Pyorala K, Fitzgerald AP, Sans S, Menotti A, De Backer G, et al Estimation of Ten-Year Risk of Fatal Cardiovascular Disease in Europe: The SCORE Project. Eur Heart J. 2003;24(11):987–1003. 10.1016/S0195-668X(03)00114-3 12788299

[4] Ridker PM, Cook NR. Statins: New American Guidelines for Prevention of Cardiovascular Disease. Lancet. 2013;382(9907):1762–5. 10.1016/S0140-6736(13)62388-0 24268611

[5] Muntner P, Colantonio LD, Cushman M, Goff DC Jr., Howard G, Howard VJ, et al Validation of the Atherosclerotic Cardiovascular Disease Pooled Cohort Risk Equations. JAMA. 2014;311(14):1406–15. 10.1001/jama.2014.2630 24682252 PMC4189930

[6] DeFilippis AP, Young R, Carrubba CJ, McEvoy JW, Budoff MJ, Blumenthal RS, et al An Analysis of Calibration and Discrimination among Multiple Cardiovascular Risk Scoresmultiple cardiovascular risk scores in a modern multiethnic cohort. Ann Intern Med. 2015;162(4):266–75. 10.7326/M14-1281 25686167 PMC4414494

[7] Kavousi M, Leening MJ, Nanchen D, Greenland P, Graham IM, Steyerberg EW, et al Comparison of Application of the ACC/AHA Guidelines, Adult Treatment Panel III Guidelines, and European Society of Cardiology Guidelines for Cardiovascular Disease Prevention in a European Cohort. JAMA. 2014;311(14):1416–23. 10.1001/jama.2014.2632 24681960

[8] Vaucher J, Marques-Vidal P, Preisig M, Waeber G, Vollenweider P. Population and Economic Impact of the 2013 ACC/AHA Guidelines Compared with European Guidelines to Prevent Cardiovascular Disease. Eur Heart J. 2014;35(15):958–9. 10.1093/eurheartj/ehu064 24569030

[9] Löwel H, Lewis M, Hörmann A, Keil U. Case Finding, Data Quality Aspects and Comparability of Myocardial Infarction Registers: Results of a South German Register Study. J Clin Epidemiol. 1991;44(3):249–60. 1999684 10.1016/0895-4356(91)90036-9

[10] Holle R, Happich M, Lowel H, Wichmann HE, Group MKS. KORA—A Research Platform for Population Based Health Research. Gesundheitswesen. 2005;67 Suppl 1:S19–25. 10.1055/s-2005-858235 16032513

[11] Erbel R, Möhlenkamp S, Moebus S, Schmermund A, Lehmann N, Stang A, et al Coronary Risk Stratification, Discrimination, and Reclassification Improvement Based on Quantification of Subclinical Coronary Atherosclerosis: The Heinz Nixdorf Recall Study. J Am Coll Cardiol. 2010;56(17):1397–406. 10.1016/j.jacc.2010.06.030 20946997

[12] Schmermund A, Lehmann N, Bielak LF, Yu P, Sheedy PF 2nd, Cassidy-Bushrow AE, et al Comparison of Subclinical Coronary Atherosclerosis and Risk Factors in Unselected Populations in Germany and US-America. Atherosclerosis. 2007;195(1):e207–16. 10.1016/j.atherosclerosis.2007.04.009 17532322 PMC2293130

[13] Schmermund A, Möhlenkamp S, Stang A, Gronemeyer D, Seibel R, Hirche H, et al Assessment of Clinically Silent Atherosclerotic Disease and Established and Novel Risk Factors for Predicting Myocardial Infarction and Cardiac Death in Healthy Middle-Aged Subjects: Rationale and Design of the Heinz Nixdorf RECALL Study. Risk Factors, Evaluation of Coronary Calcium and Lifestyle. Am Heart J. 2002;144(2):212–8. 10.1067/mhj.2002.123579 12177636

[14] Janssen KJ, Moons KG, Kalkman CJ, Grobbee DE, Vergouwe Y. Updating Methods Improved the Performance of a Clinical Prediction Model in New Patients. J Clin Epidemiol. 2008;61(1):76–86. 10.1016/j.jclinepi.2007.04.018 18083464

[15] Harrell FE Jr., Califf RM, Pryor DB, Lee KL, Rosati RA. Evaluating the Yield of Medical Tests. JAMA. 1982;247(18):2543–6. 10.1001/jama.1982.03320430047030 7069920

[16] Harrell FE Jr., Lee KL, Califf RM, Pryor DB, Rosati RA. Regression Modelling Strategies for Improved Prognostic Prediction. Stat Med. 1984;3(2):143–52. 10.1002/sim.4780030207 6463451

[17] Steyerberg EW, Vickers AJ, Cook NR, Gerds T, Gonen M, Obuchowski N, et al Assessing the Performance of Prediction Models: a Framework for Traditional and Novel Measures. Epidemiology. 2010;21(1):128–38. 10.1097/EDE.0b013e3181c30fb2 20010215 PMC3575184

[18] Crowson CS, Atkinson EJ, Therneau TM. Assessing Calibration of Prognostic Risk Scores. Stat Methods Med Res. 2014 Epub 2013/08/03. PubMed Central PMCID: PMC3933449. 10.1177/0962280213497434 23907781 PMC3933449

[19] R Core Team. R: A Language and Environment for Statistical Computing. R Foundation for Statistical Computing; 2014 https://www.R-project.org/.

[20] Robin X, Turck N, Hainard A, Tiberti N, Lisacek F, Sanchez JC, et al pROC: An Open-Source Package for R and S+ to Analyze and Compare ROC Curves. BMC Bioinformatics. 2011;12:77 10.1186/1471-2105-12-77 21414208 PMC3068975

[21] Therneau TM. A Package for Survival Analysis in S. R package version 2.37–7. 2014.

[22] Schröder MS, Culhane AC, Quackenbush J, Haibe-Kains B. survcomp: An R/Bioconductor Package for Performance Assessment and Comparison of Survival Models. Bioinformatics. 2011;27(22):3206–8. 10.1093/bioinformatics/btr511 21903630 PMC3208391

[23] Hense HW, Schulte H, Lowel H, Assmann G, Keil U. Framingham Risk Function Overestimates Risk of Coronary Heart Disease in Men and Women from Germany—Results from the MONICA Augsburg and the PROCAM Cohorts. Eur Heart J. 2003;24(10):937–45. 12714025 10.1016/s0195-668x(03)00081-2

[24] Menotti A, Puddu PE, Lanti M. Comparison of the Framingham Risk Function-based Coronary Chart with Risk Function from an Italian Population Study. Eur Heart J. 2000;21(5):365–70. 10.1053/euhj.1999.1864 10666350

[25] Brindle P, Emberson J, Lampe F, Walker M, Whincup P, Fahey T, et al Predictive Accuracy of the Framingham Coronary Risk Score in British Men: Prospective Cohort Study. BMJ. 2003;327(7426):1267 10.1136/bmj.327.7426.1267 14644971 PMC286248

[26] Cooney MT, Dudina AL, Graham IM. Value and Limitations of Existing Scores for the Assessment of Cardiovascular Risk: a Review for Clinicians. J Am Coll of Cardiol. 2009;54(14):1209–27. 10.1016/j.jacc.2009.07.020 19778661

[27] Thygesen K, Alpert JS, Jaffe AS, Simoons ML, Chaitman BR, White HD, et al Third Universal Definition of Myocardial Infarction. Circulation. 2012;126(16):2020–35. 10.1161/CIR.0b013e31826e1058 22923432

[28] Committee on Preventing the Global Epidemic of Cardiovascular Disease: Meeting the Challenges in Developing Countries; Institute of Medicine (US). Epidemiology of Cardiovascular Disease. In: Promoting Cardiovascular Health in the Developing World: A Critical Challenge to Achieve Global Health. 1 ed. Washington (DC), USA: The National Academies Press; 2010. 20945571

[29] Müller-Nordhorn J, Binting S, Roll S, Willich SN. An Update on Regional Variation in Cardiovascular Mortality within Europe. Eur Heart J. 2008;29(10):1316–26. 10.1093/eurheartj/ehm604 18256043

[30] Müller-Nordhorn J, Rossnagel K, Mey W, Willich SN. Regional Variation and Time Trends in Mortality from Ischaemic Heart Disease: East and West Germany 10 Years after Reunification. J Epidemiol Community Health. 2004;58(6):481–5. 15143116 10.1136/jech.2003.013367 PMC1732785

[31] Stang A, Stang M. An Inter-State Comparison of Cardiovascular Risk Factors in Germany: Towards an Explanation of High Ischemic Heart Disease Mortality in Saxony-Anhalt. Deutsches Ärzteblatt International. 2014;111(31–32):530–6. 10.3238/arztebl.2014.0530 25145511 PMC4148713

[32] Assmann G, Schulte H, Cullen P, Seedorf U. Assessing Risk of Myocardial Infarction and Stroke: New Data from the Prospective Cardiovascular Münster (PROCAM) Study. Eur J Clin Invest. 2007;37(12):925–32. 10.1111/j.1365-2362.2007.01888.x 18036028

