# Supplementary material for: Recalibration of the ACC/AHA Risk Score in Two Population-Based German Cohorts
Source: PLoS One. 2016 Oct 12;11(10):e0164688. doi: 10.1371/journal.pone.0164688 (PMC5061315; doi:10.1371/journal.pone.0164688)
Supplement: S1 Appendix — (PDF) [file pone.0164688.s001.pdf]

Drop-out analysis

Methods

In KORA, 486 subjects were lost-to-follow-up without information on incident ASCVD. To rule out possible bias in analysing the ACC/AHA risk score, we performed a sensitivity analysis, analysing the risk profile of these subjects and imputing their missing event status. For this, we used a stratified bootstrapping resampling technique (number of bootstrap samples: b = 300) and three imputation scenarios, randomly assigning events with event rate 50%, 20% or 10%. Since we did not have information on survival or censoring time for these subjects, we used the area under the receiver operating characteristic curve (AUC) as a measure of discrimination performance in this sensitivity analysis, as it does not incorporate survival times.[1] We then investigated the impact of the three event rate scenarios on the performance of the ACC/AHA risk score.

Results

Exclusion of subjects with missing information on risk score variables resulted in 5,238 completely observed subjects and 464 (8%) subjects with missing outcome information. Out of seven risk factors included in the ACC/AHA risk equation, we observed significant differences regarding five risk factors (age, total cholesterol, systolic blood pressure, smoking and diabetes) between KORA subjects without follow-up on incident ASCVD events and KORA subjects included in the main analysis. Using the ACC/AHA risk equations, mean estimated 10 year ASCVD risk was greater for KORA subjects without follow-up (11.5%) than for KORA subjects with follow-up (10.0%). Details are depicted in S2 Table.

Results from imputing missing outcome information with event rate 10%, which was closest to the ASCVD risk estimated by the ACC/AHA risk score, showed a calibration performance comparable to the analysis of the completely observed data, however, poorer performance

regarding discriminative power (Complete data: AUC = 0.78 [0.76, 0.81], imputation with 10% event rate: AUC = 0.76 [0.73, 0.78]). Imputation with event rate 20% and 50% improved calibration of the ACC/AHA risk score in KORA, however, with considerably reduced discrimination ability (event rate 20%: AUC = 0.74 [0.72, 0.76]; event rate 50%: AUC = 0.70 [0.68, 0.72]). Results are depicted in S3 Fig.

[1] Hanley JA, McNeil BJ. The meaning and use of the area under a receiver operating characteristic (ROC) curve. Radiology. 1982;143(1):29-36.

S2 Table. Risk profile of KORA subjects with and without follow-up on incident ASCVD events.

	KORA		
**Characteristic[a]**	**With follow-up**	**Without follow-up**	**p-value[b]**
**N[c] (%)**	5238	464[d]	
**Age[e] (years)**	55.9 +- 9.7	57.6 +- 10.2	<0.001
**Sex male**	49.2 (2623)	44.4 (216)	0.051
**Total cholesterol (mg/dL)**	234.2 (207.9, 263.7)	240.4 (212.8, 270.4)	0.016
**HDL[f] cholesterol (mg/dL)**	53.3 (43.8, 65.9)	53.0 (44, 64.3)	0.617
**Systolic blood pressure (mmHg)**	132.0 (120.5, 146.5)	135.5 (123.5, 148)	<0.01
**Intake of antihypertensive drugs**	20.4 (1085)	23.2 (112)	0.159
**Intake of statins or fibrates**	5.1 (270)	6.8 (33)	0.118
**Current smoker**	21.1 (1122)	25.1 (122)	0.043
**Diabetes**	4.8 (257)	7.2 (35)	0.028
**Average 10 year ASCVD risk (%) according to the ACC/AHA risk score**	10.0 ± 10.2	11.5 ± 10.5	<0.001

[a]Depicted are absolute numbers (percentage) for categorical and median (first quartile, third quartile) for continuous variables.

[b]p-value from Kruskal-Wallis test for continuous and Chi-square-test for categorical variables.

[c]N = Sample size.

[d]Of n=486 subjects without follow-up, 22 subjects had to be excluded due to missing information in one or more risk factors.

[e]Age (years) is shown as mean ± standard deviation.

[f]HDL = High-density lipoprotein.

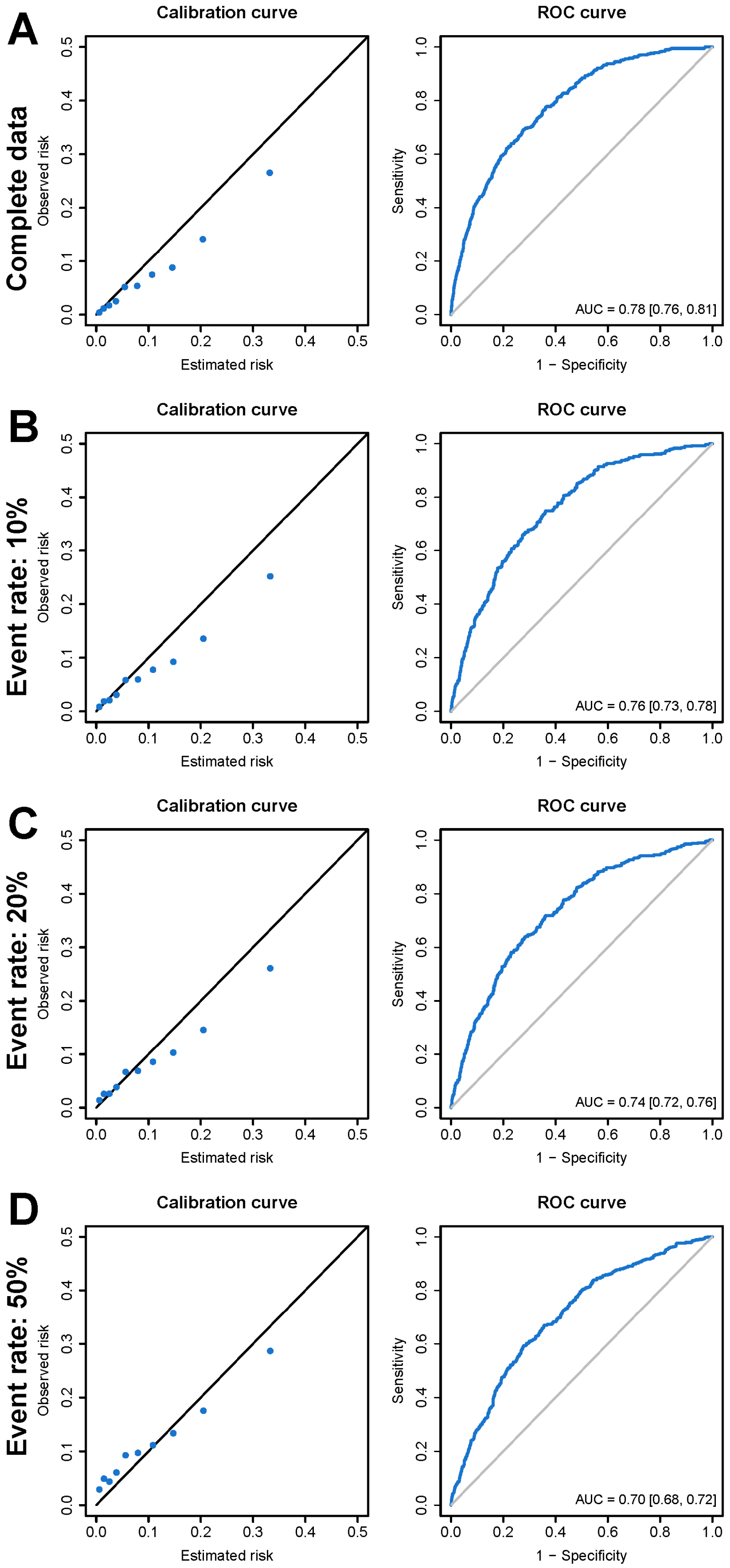

S3 Fig. Calibration and ROC curve from drop-out analysis. ROC analysis and calibration performance of ACC/AHA risk score after imputation of lost-to-follow-up subjects in KORA: Complete case analysis without imputation (part A), random assignment of event status to lost-to-follow-up subjects with event-rate 10% (part B), 20% (part C) and 50% (part D).
