# Supplementary figures and images for: Recalibration of the ACC/AHA Risk Score in Two Population-Based German Cohorts
Source: PLoS One. 2016 Oct 12;11(10):e0164688. doi: 10.1371/journal.pone.0164688 (PMC5061315; doi:10.1371/journal.pone.0164688)

## KORA S3 & S4

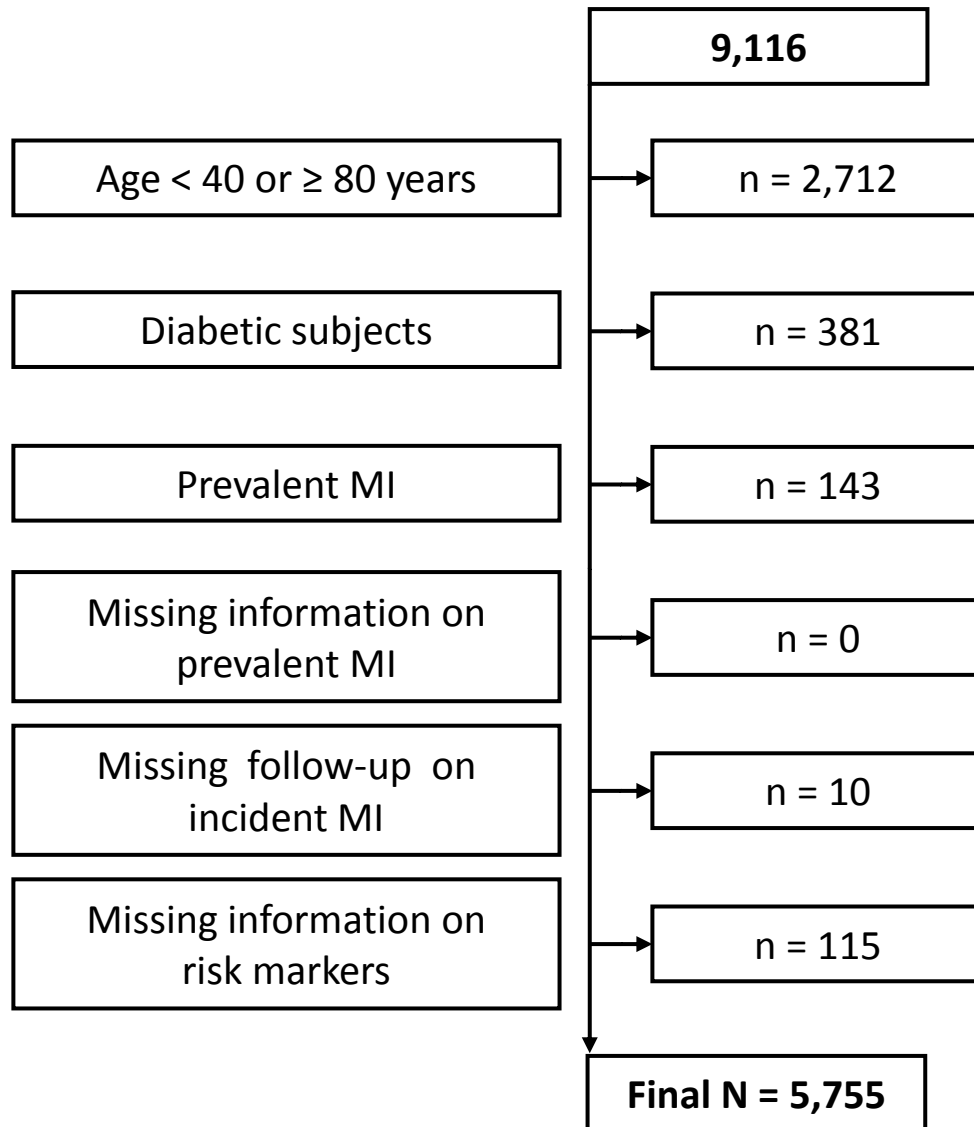

## Heinz Nixdorf Recall

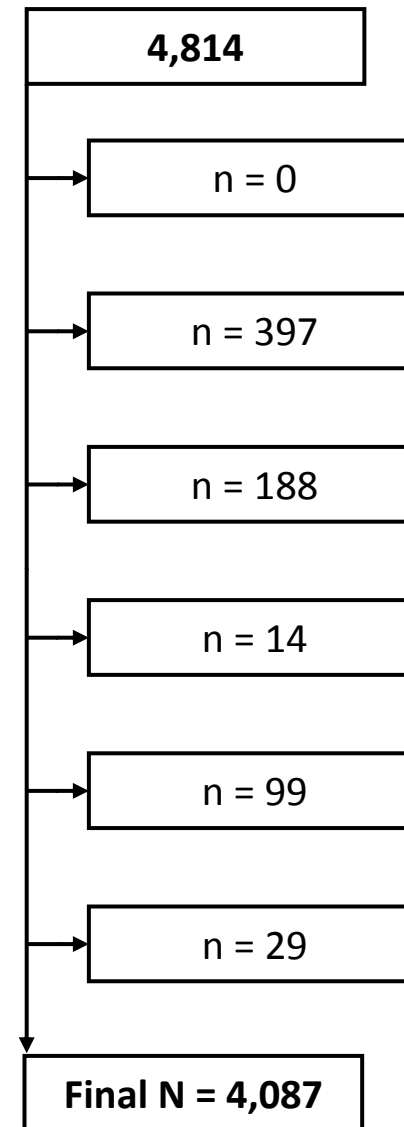

Supplement: S1 Fig — (PDF) [file pone.0164688.s002.pdf]

### ACC/AHA risk score

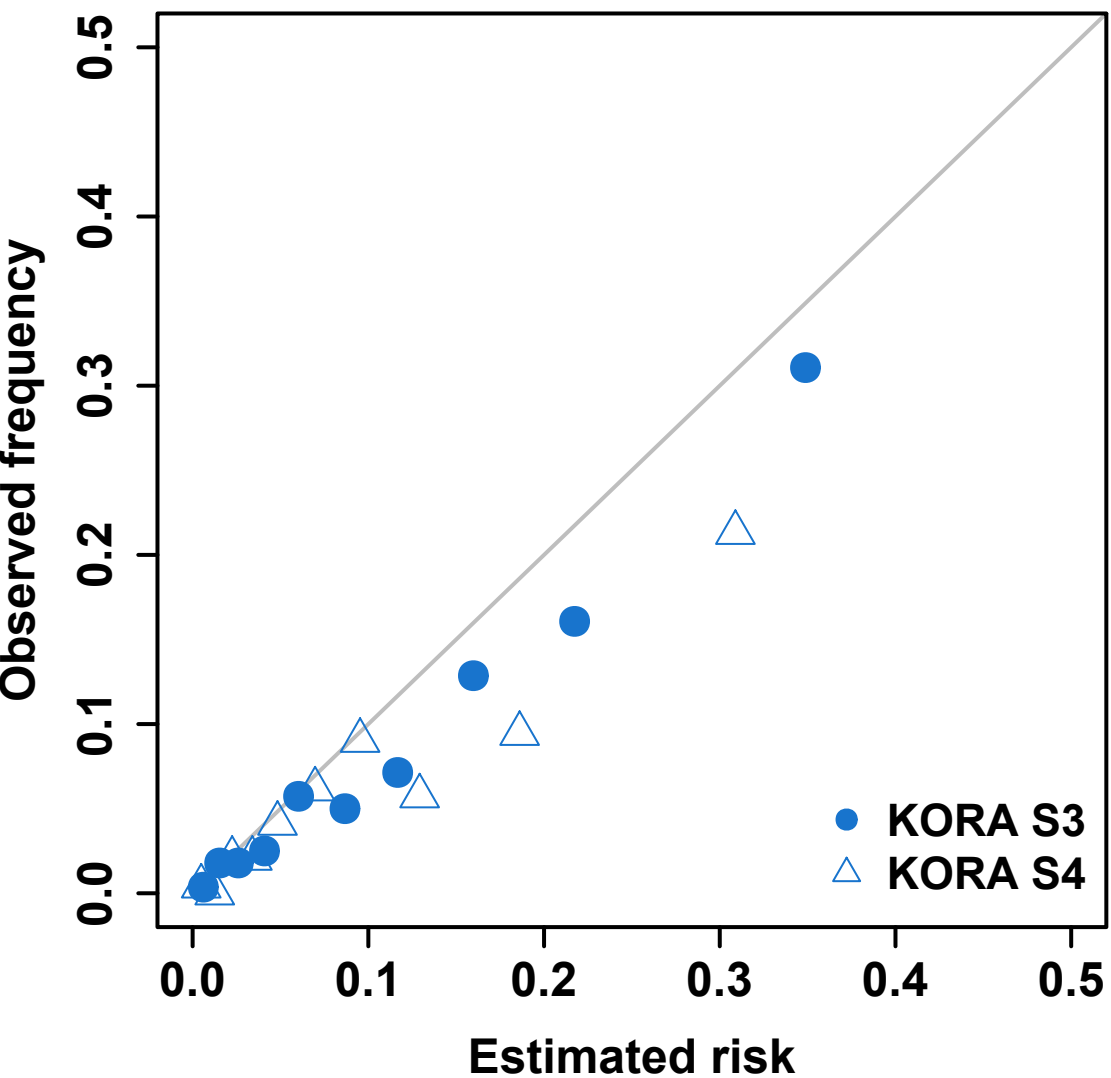

### Recalibrated ACC/AHA risk score

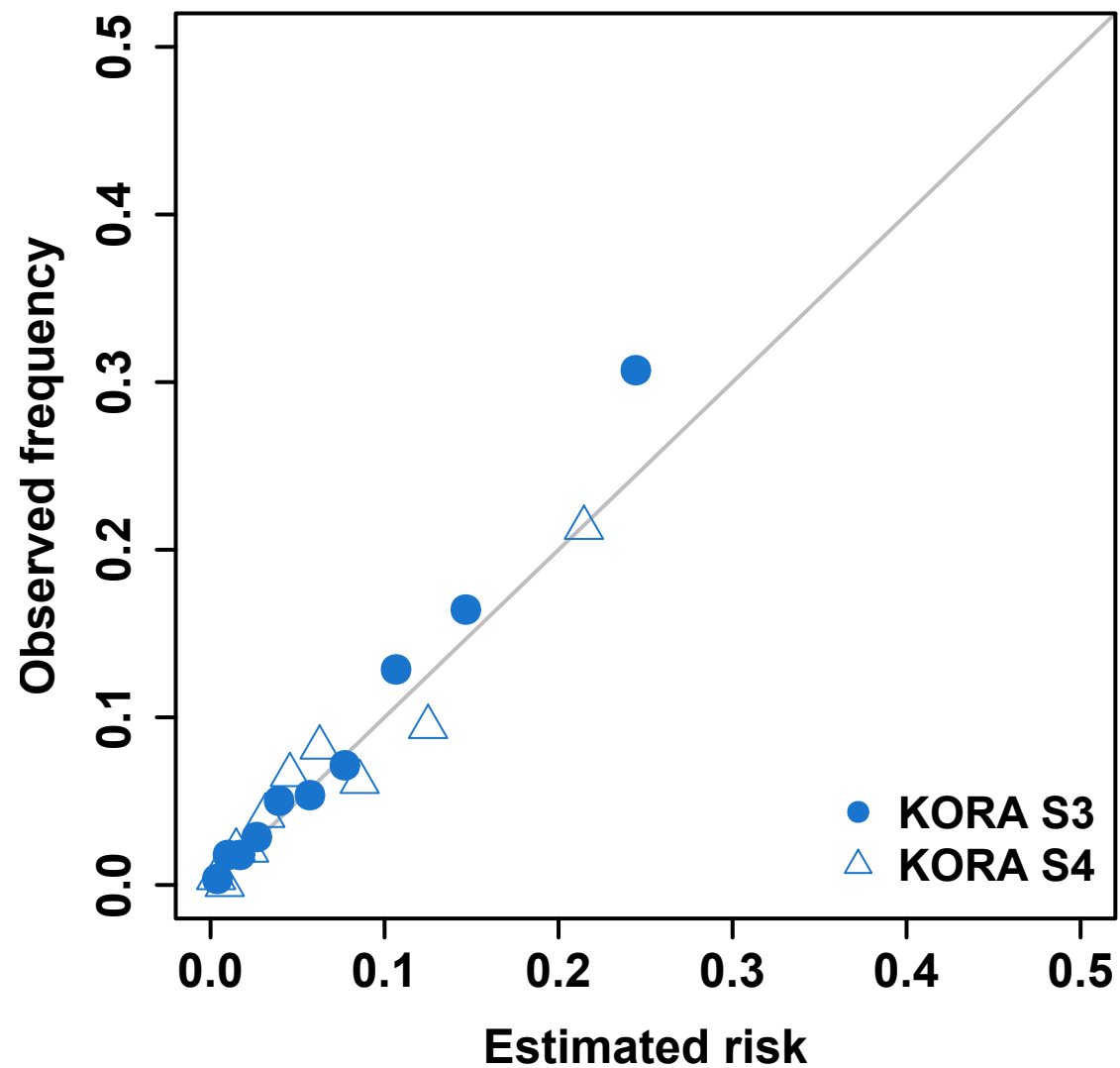

Supplement: S2 Fig — Calibration plot of the ACC/AHA risk score before (left) and after (right) recalibration separately for KORA S3 (filled circles) and KORA S4 (triangles). (PDF) [file pone.0164688.s003.pdf]
