# Supplementary material for: Recalibration of the ACC/AHA Risk Score in Two Population-Based German Cohorts
Source: PLoS One. 2016 Oct 12;11(10):e0164688. doi: 10.1371/journal.pone.0164688 (PMC5061315; doi:10.1371/journal.pone.0164688)
Supplement: S1 Table — (PDF) [file pone.0164688.s004.pdf]

Part A: Originally published coefficients of ACC/AHA risk score for Caucasian race
(Goff et al. 2014)

	**Women**	**Men**
Ln Age (years)	-29.799	12.344
Ln Age (years), Squared	4.884	0
Ln Total Cholesterol (mg/dL)	13.54	11.853
Ln HDL Cholesterol (mg/dL)	-13.578	-7.99
Current Smoker (1=yes, 0=no)	7.574	7.837
Diabetes (1=yes, 0=no)	0.661	0.658
Ln Age x Ln Total Cholesterol	-3.114	-2.664
Ln Age x Ln HDL Cholesterol	3.149	1.769
Ln Untreated SBP (mm Hg)	1.957	1.764
Ln Treated SBP (mm Hg)	2.019	1.797
Ln Age x Current Smoker	-1.665	-1.795
Population mean	-29.18	61.18
Baseline Survival at 10 years	0.9665	0.9144

Ln = natural logarithm, HDL = High-density lipoprotein, SBP = Systolic blood pressure.

Part B: Calculation of ASCVD 10 year risk for German population with and without recalibration of ACC/AHA risk score

- ASCVD risk by <u>original</u> ACC/AHA risk equations:
  10 year risk of ASCVD = 1 - Baseline Survival at 10 years $^{\text{exp(Riskscore - Population mean)}}$

- ASCVD risk using the <u>recalibrated</u> ACC/AHA risk equations:
  correction factor = $\ln\left(\frac{\frac{observed\ event\ frequency}{1-observed\ event\ frequency}}{\frac{mean\ predicted\ risk}{1-mean\ predicted\ risk}}\right) = \ln\left(\frac{\frac{0.06923566}{0.93076434}}{\frac{0.1030771}{0.8969229}}\right) = -0.434997$
  10 year risk of ASCVD = 1 - Baseline Survival at 10 years $^{\text{exp (Riskscore - Population mean - 0.434997)}}$
